# Application of Complementary and Alternative Medicine on Neurodegenerative Disorders: Current Status and Future Prospects

**DOI:** 10.1155/2012/930908

**Published:** 2012-12-02

**Authors:** Paul Siu-Po Ip, Karl Wah-Keung Tsim, Kelvin Chan, Rudolf Bauer

**Affiliations:** ^1^School of Chinese Medicine, The Chinese University of Hong Kong, Hong Kong; ^2^Division of Life Science, The Hong Kong University of Science and Technology, Hong Kong; ^3^Faculty of Pharmacy, The University of Sydney, Sydney, NSW 2006, Australia; ^4^Centre for Complementary Medicine Research, University of Western Sydney, Sydney, NSW 2560, Australia; ^5^Department of Pharmacognosy, Institute of Pharmaceutical Sciences, University of Graz, 8010 Graz, Austria

Neurodegenerative disorders are defined as hereditary and sporadic conditions which are characterized by progressive loss of structure or function of neurons in sensory, motor, and cognitive systems. Alzheimer's disease, Parkinson's disease, and depression are well-known examples of neurodegenerative disorders. The World Health Organization estimates that, by 2040, neurodegenerative diseases will surpass cancer as the principal cause of death in industrialized countries. Despite various advances in the understanding of the diseases, pharmacological treatment by conventional medicine has not obtained satisfactory results. Therefore, complementary and alternative medicine (CAM) can be a potential candidate for the preventative treatment of the disorders. The aim of this special issue is to demonstrate the clinical evidence and explore the acting mechanisms of CAM in treating neurodegenerative disorders. 

Alzheimer's disease is a well-recognized neurodegenerative disease characterized by a progressive deterioration of cognitive function and memory. At present, there are no effective treatments that can stop or reverse the progression of the disease. Pharmaceutical interventions that aim to delay the deterioration of this disease have been extensively studied. Although cholinesterase inhibitors and an N-methyl-D-aspartate receptor antagonist have been widely used for treating the syndromes of the disease, these drugs have not shown promising results and their uses are always limited by their undesirable side effects. In this special issue, several studies have shown that CAM can be useful for the management of the disease. 

Although the pathological cause of Alzheimer's disease has not been fully understood, the deposition of beta-amyloid is believed to be one of the risk factors. Therefore, neurotoxicity induced by beta-amyloid is commonly used as a cellular or animal model of Alzheimer's disease. In this issue, an animal study showed that oral administration of Yi-Chi-Tsung-Ming-Tang (Table 1) ameliorated beta-amyloid injection-induced learning and memory impairments. Further investigation by biochemical analysis showed that the herbal decoction decreased amyloid accumulation and reversed acetylcholine decline in the hippocampus of the animals treated with beta-amyloid. 

There are two studies on cellular model of Alzheimer's disease with *Flemingia macrophylla *and *Uncaria rhynchophylla*, respectively. Beneficial effects of both medical herbs have been suggested for the management of Alzheimer's disease. By using bioassay-guided fractionation, rhynchophylline and isorhynchophylline have been identified as the active ingredients of *Uncaria rhynchophylla*. The neuroprotective effect of these chemical ingredients has been suggested to be mediated by inhibiting intracellular calcium overloading and tau protein hyperphosphorylation.

Depression is a chronic mental disorder clinically characterized by a pervasive low mood, loss of interest or pleasure in daily activities, low self-esteem, and a high suicidal tendency. Although the monoamine theory of depression has been extensively investigated, it is unable to fully explain the pathophysiology of depression. In recent years, a huge amount of evidences suggesting a causal relationship between the incidence of major depressive disorders and neurodegenerative processes such as the decreased neurotrophic factors, altered neuronal plasticity, neuronal atrophy, or destruction in the hippocampus and cortex has been published. 

At present, there are several types of antidepressants available for pharmaceutical management of the disease including tricyclic antidepressants, monoamine oxidase inhibitors, selective serotonin reuptake inhibitors, noradrenergic reuptake inhibitors, serotonin, and noradrenaline reuptake inhibitors. However, due to the multiple pathogenic factors involved in depression, many antidepressant drugs show low response rates and may cause adverse side effects such as cardiotoxicity, hypertensive crisis, sexual dysfunction, and sleep disorder. Therefore, a number of herbal remedies have been suggested to be safe, better tolerated, and efficacious antidepressants. In this issue, several research articles have shown that herbal prescriptions, including Shu-Yu-San, Kai-Xin-San, Baihe-Dihuang-Tang, and Danggui-Shaoyao-San (Table 1), are effective antidepressants on animal model of depression. 

In this special issue, we have collected a couple of clinical studies on the application of CAM in treating neurodegenerative diseases. Although the scale of these studies is small, all of them have demonstrated a promising effect of CAM on neurodegenerative diseases. For example, a study of Toki-Shakuyaku-San (Danggui- Shaoyao-San in Chinese phonetic name), a six-herb Chinese medicine (Table 1), on patients with mild cognitive impairment and Alzheimer's disease showed that treatment with Toki-Shakuyaku-San for eight weeks significantly increased regional cerebral blood flow in the posterior cingulate and tended to improve cognitive impairment in these patients. Another randomized clinical trial showed that Saffron (flower of *Crocus sativus*) supplement improved retinal flicker sensitivity in patients with early age-related macular degeneration and the beneficial effect of the herbal drug was extended over a 14-month follow-up study. 

In this special issue, a large amount of evidences have shown that CAM can be an efficacious treatment for neurodegenerative disorders. However, a large-scale, double-blind, and placebo-controlled trial is still needed to demonstrate the clinical effect of CAM on these diseases.

## Figures and Tables

**Table 1:** Composition of herbal formulae.

Herbal formulae	Component herbs^#^
Yi-Chi-Tsung-Ming-Tang	Astragali Radix, Cimicifugae Rhizoma, Ginseng Radix, Glycyrrhizae Radix et Rhizoma, Paeoniae Radix Alba, Phellodendri Chinensis Cortex, Puerariae Lobatae Radix, and Viticis Fructus.

Shu-Yu-San	Albiziae Flos, Acori Tatarinowii Rhizoma, Bupleuri Radix, Curcumae Radix, Gardeniae Fructus, Menthae Herba, Polygalae Radix, Poria, and Ziziphi Spinosae Semen.

Kai-Xin-San	Acori Tatarinowii Rhizoma, Ginseng Radix et Rhizome, Polygalae Radix, and Poria.

Baihe-Dihuang-Tang	Lilii Bulbus and Rehmanniae Radix.

Danggui-Shaoyao-San (Toki-Shakuyaku-San)	Alismatis Rhizoma, Angelicae Sinensis Radix, Atractylodis Macrocephalae Rhizoma Chuanxiong Rhizoma, Paeoniae Radix Alba, and Poria.

^#^Official name listed in Pharmacopoeia of China (2010 Edition), Chinese Medical Science and Technology Press, Beijing, China.

